# Discovery of New Genes Involved in Curli Production by a Uropathogenic Escherichia coli Strain from the Highly Virulent O45:K1:H7 Lineage

**DOI:** 10.1128/mBio.01462-18

**Published:** 2018-08-21

**Authors:** Nguyen Thi Khanh Nhu, Minh-Duy Phan, Kate M. Peters, Alvin W. Lo, Brian M. Forde, Teik Min Chong, Wai-Fong Yin, Kok-Gan Chan, Milan Chromek, Annelie Brauner, Matthew R. Chapman, Scott A. Beatson, Mark A. Schembri

**Affiliations:** a School of Chemistry and Molecular Biosciences, the University of Queensland, Brisbane, Queensland, Australia; b Australian Infectious Diseases Research Centre, the University of Queensland, Brisbane, Queensland, Australia; c Australian Centre for Ecogenomics, the University of Queensland, Brisbane, Queensland, Australia; d Division of Genetics and Molecular Biology, Institute of Biological Sciences, Faculty of Sciences, University of Malaya, Kuala Lumpur, Malaysia; e International Genome Centre, Jiangsu University, Zhenjiang, China; f Department of Microbiology, Tumor and Cell Biology, Division of Clinical Microbiology, Karolinska Institutet and Karolinska University Hospital, Stockholm, Sweden; g Department of Pediatrics, CLINTEC, Karolinska University Hospital and Karolinska Institutet, Stockholm, Sweden; h Department of Molecular, Cellular, and Developmental Biology, University of Michigan, Ann Arbor, Michigan, USA; The Ohio State University School of Medicine

**Keywords:** Escherichia coli, biofilms, curli, urinary tract infection, virulence, virulence regulation

## Abstract

Curli are bacterial surface-associated amyloid fibers that bind to the dye Congo red (CR) and facilitate uropathogenic Escherichia coli (UPEC) biofilm formation and protection against host innate defenses. Here we sequenced the genome of the curli-producing UPEC pyelonephritis strain MS7163 and showed it belongs to the highly virulent O45:K1:H7 neonatal meningitis-associated clone. MS7163 produced curli at human physiological temperature, and this correlated with biofilm growth, resistance of sessile cells to the human cationic peptide cathelicidin, and enhanced colonization of the mouse bladder. We devised a forward genetic screen using CR staining as a proxy for curli production and identified 41 genes that were required for optimal CR binding, of which 19 genes were essential for curli synthesis. Ten of these genes were novel or poorly characterized with respect to curli synthesis and included genes involved in purine *de novo* biosynthesis, a regulator that controls the Rcs phosphorelay system, and a novel repressor of curli production (referred to as *rcpA*). The involvement of these genes in curli production was confirmed by the construction of defined mutants and their complementation. The mutants did not express the curli major subunit CsgA and failed to produce curli based on CR binding. Mutation of *purF* (the first gene in the purine biosynthesis pathway) and *rcpA* also led to attenuated colonization of the mouse bladder. Overall, this work has provided new insight into the regulation of curli and the role of these amyloid fibers in UPEC biofilm formation and pathogenesis.

## IMPORTANCE

Uropathogenic Escherichia coli (UPEC) strains are the most common cause of urinary tract infection, a disease increasingly associated with escalating antibiotic resistance. UPEC strains possess multiple surface-associated factors that enable their colonization of the urinary tract, including fimbriae, curli, and autotransporters. Curli are extracellular amyloid fibers that enhance UPEC virulence and promote biofilm formation. Here we examined the function and regulation of curli in a UPEC pyelonephritis strain belonging to the highly virulent O45:K1:H7 neonatal meningitis-associated clone. Curli expression at human physiological temperature led to increased biofilm formation, resistance of sessile cells to the human cationic peptide LL-37, and enhanced bladder colonization. Using a comprehensive genetic screen, we identified multiple genes involved in curli production, including several that were novel or poorly characterized with respect to curli synthesis. In total, this study demonstrates an important role for curli as a UPEC virulence factor that promotes biofilm formation, resistance, and pathogenesis.

## INTRODUCTION

The bacterial biofilm lifestyle is integral to many infections and promotes persistence, protection from host innate immune factors, and resistance to antibiotics. Uropathogenic Escherichia coli (UPEC) strains, the major cause of human urinary tract infection (UTI) (1–3), form biofilms on catheters (4–6) and in the bladder, either on the surface of the epithelium or within superficial facet cells in the form of intracellular bacterial communities (IBCs) (7–11). UPEC biofilm formation is mediated by multiple factors, including adhesins such as fimbriae, curli, and autotransporter proteins (12–16), and polysaccharides such as the capsule and cellulose (12, 17).

Curli are amyloid fibers that form a key component of the UPEC extracellular biofilm matrix (18–20). They are also highly proinflammatory (21), induce the formation of immunogenic complexes with DNA to stimulate autoimmune responses (22), and can neutralize human cathelicidin (LL-37), a soluble antimicrobial peptide that protects against UTI (23, 24). Curli synthesis is coordinated via a type VIII secretion system also known as the nucleation-precipitation pathway (25) and requires the expression of seven genes from two divergent operons (*csgBAC* and *csgDEFG*) (25, 26). The *csgBAC* operon encodes the curli major subunit protein CsgA, the curli nucleator protein CsgB, and the periplasmic chaperone CsgC. The *csgDEFG* operon encodes CsgD, a regulator that controls the transcription of curli genes, CsgG, an outer membrane secretion pore essential for curli biogenesis, and CsgEF, curli accessory factors (27–29). Curli production is often linked to the synthesis of cellulose as an additional component of the biofilm matrix. Recent work has shown that E. coli cells produce a chemically modified phosphoethanolamine cellulose (30), and this polysaccharide can dampen curli-stimulated immune induction (23, 31).

The control of curli biosynthesis is tightly coordinated by a complex signaling network that involves CsgD and several other regulators (32). The sigma factor RpoS, which controls gene expression during stationary-phase growth and stress, is a positive regulator of curli production (33, 34). Another transcriptional response regulator, MlrA, binds directly to the *csgD* promoter and positively regulates curli production in an RpoS-dependent manner (35, 36). Other regulators that control curli production include the osmotic sensor-response pair OmpR-EnvZ (37, 38), the envelope stress sensor-response pair CpxR-CpxA (37, 39), the Rcs phosphorelay system (40, 41), the histone-like nucleoid-structuring (H-NS) protein (33, 34, 42), and integration host factor (IHF) (33, 34, 42). Curli expression is also activated by the second messenger cyclic-di-GMP, which directly enhances transcription of the *csgD* regulatory gene (43–46).

Most of our knowledge on curli regulation and biosynthesis comes from studies on E. coli K-12 strains at growth temperatures below 30°C, and an understanding of their genetic control and contribution to pathogenesis in UPEC at human physiological temperature is lacking. Given that many UPEC strains produce curli at 37°C during human infection (23, 47), we investigated the role of these amyloid fibers in the UPEC pyelonephritis strain MS7163, an LL-37-resistant isolate identified in a previous study (24). We showed that MS7163 produces curli, but not cellulose, and sequencing of its genome revealed the genetic basis of this phenotype. We also demonstrated that curli mediate resistance of sessile cells to LL-37 during MS7163 biofilm growth and enhance colonization of the mouse bladder. The production of curli by MS7163 resulted in binding to the dye Congo red (CR), and we exploited this specific property in combination with transposon mutagenesis and transposon-directed insertion site sequencing (TraDIS) to identify genes involved in curli biogenesis during growth at 37°C. Our screen identified several novel genes, including genes involved in purine *de novo* biosynthesis and two new regulators, all of which were confirmed to affect curli production through the generation of specific mutants and complementation. Finally, the importance of these genes to UPEC virulence was demonstrated in a mouse UTI model.

## RESULTS

### MS7163 produces curli during growth at 37°C and binds Congo red.

**FIG 1:**
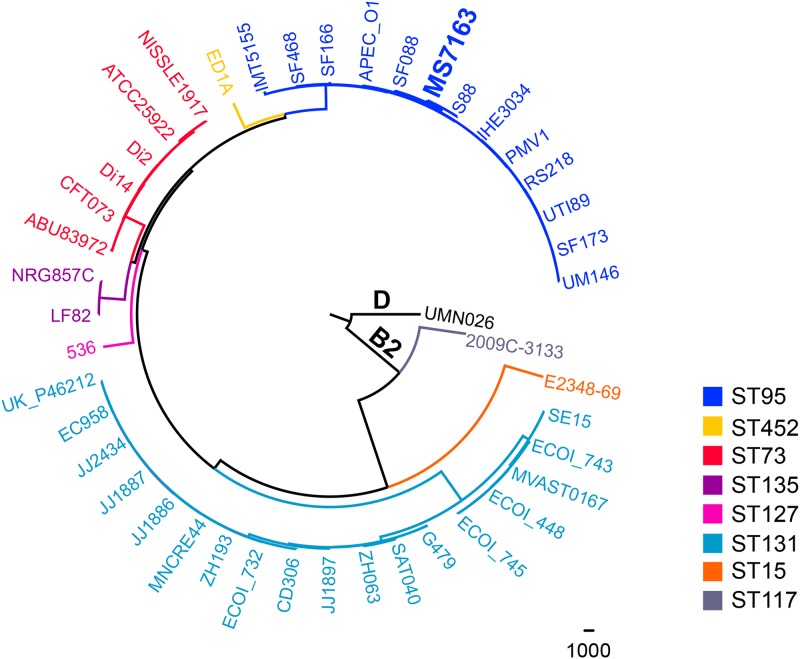
Phylogenetic relationship of MS7163 and other E. coli phylogenetic group B2 strains. The maximum likelihood phylogenetic tree of MS7163 and other strains from E. coli phylogroup B2 (listed in Table S2) was reconstructed using kSNP with a k-mer size of 21 and rooted using the E. coli phylogroup D strain UMN026 as an outgroup. Branches were colored according to their ST types (Achtman MLST scheme). MS7136 (boldface) clustered into the same group of E. coli ST95 and was closely related to NMEC strain S88.

**FIG 2:**
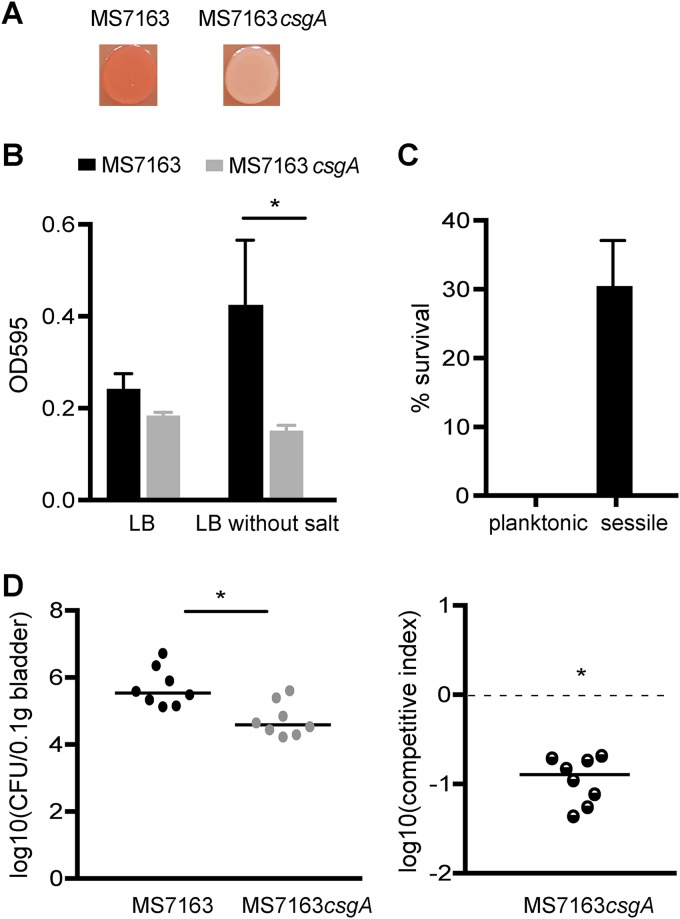
Curli enhance UPEC biofilm formation and virulence. (A) CR staining of wt MS7163 and MS7163*csgA*. (B) Biofilm formation between the wt MS7163 and the curli knockout mutant MS7163*csgA* in LB and LB without salt. An asterisk represents a *P* value of <0.0001 for a *t* test between MS7163 and MS7163*csgA*; error bars represent standard deviation from three independent replicates. (C) Survival rates between the planktonic and sessile cells after 2 h of incubation in the presence of 64 µg/ml LL-37. Error bars represent standard deviation from three independent replicates. (D) Competitive mixed infection mouse experiment employing a 1:1 mixture of MS7163 and MS7163*csgA*. (Left panel) Total CFU enumerated from the bladder of infected mice. Each symbol represents the CFU for each mouse, and the median is represented by a horizontal line. (Right panel) Competitive fitness index of bladder colonization. Each symbol represents data from an individual mouse in bladder tissue at 24 h postinfection. Logarithmic values of competitive indices are plotted on the *y* axis; horizontal bars represent median values. A log_10_ fitness index below 0 (shown by the dashed line) indicates that MS7163*csgA* was at a competitive disadvantage. Statistical significance was determined by the two-tailed Wilcoxon’s signed rank test (*, *P* < 0.05).

MS7163 is a curli-producing UPEC pyelonephritis strain resistant to LL-37 (24). In line with this phenotype, MS7163 bound strongly to CR following growth at both 37°C and 28°C on YESCA-CR agar (see Materials and Methods). Complete genome sequencing of MS7163 revealed it belongs to the E. coli B2 phylogenetic group and multilocus sequence type 95 (ST95). The MS7163 genome comprises a chromosome containing 5,074,586 nucleotides and two large plasmids (pMS7163A, 133,843 bp; pMS7163B, 84,078 bp) (see Fig. S1, Fig. S2, and Data Set S1 in the supplemental material). Whole-genome phylogenetic analysis of MS7163 and other completely sequenced B2 strains (listed in Data Set S2 in the supplemental material) revealed MS7163 clusters in a clade with the reference ST95 strains UTI89, a cystitis strain (48), and APEC-O1, an avian-pathogenic E. coli strain (49), and in a subclade with the neonatal meningitis E. coli (NMEC) strain S88 (50) (Fig. 1). Because curli production and CR binding by E. coli are frequently associated with the synthesis of cellulose, we examined the MS7163 genes involved in cellulose regulation and biosynthesis. MS7163 possessed a mutation in the essential cellulose production gene *bcsA* (with a G deletion at nucleotide 556 of *bcsA* causing its premature termination), leading us to predict that it is unable to produce cellulose. To demonstrate this, we mutated the *csgA* major curli subunit gene (to generate the mutant MS7163*csgA*) and examined the phenotypes of MS7163 and MS7163*csgA* on YESCA-CR agar. In contrast to wild-type (wt) MS7163, MS7163*csgA* did not bind CR, confirming that the production of curli (and not cellulose) is specifically associated with this phenotype (Fig. 2A).

10.1128/mBio.01462-18.2 FIG S1  Comparative genomic analysis of the MS7163 chromosome and other sequenced E. coli strains. (A) BRIG visualization based on BLASTN search of the MS7163 chromosome and other sequenced E. coli phylogroup B2 strains, highlighting the distribution and variability of MS7163 prophages and genomic islands. Seven MS7163 prophages (phi1 to phi7) and nine genomic islands (GI-*aspV*, GI-*thrW*, GI-*icdA*, GI-*asnT*–high-pathogenicity island [HPI], GI-*asnW*, GI-*metV*, GI-*pheV*, GI-*pheU*, and GI-*leuX*) are indicated as the outermost green and blue boxes, respectively. The two innermost rings represent the GC content (black) and GC skew (green/purple) of MS7163. The remaining circles indicate BLASTN searches of E. coli strains against the MS7163 chromosome, with the nucleotide conservation cutoff set at 70%. Strains are color coded according to their ST and arranged from the inner to outer rings as follows. ST95 strains S88, SF-88, APEC O1, UTI89, IHE3034, PMV-1, RS218, SF-173, UMN146, APEC IMT5155, SF-468, and SF-166 are in blue. ST452 strain ED1a is in yellow. ST73 strains Nissle 1917, ATCC 25922, clone D i2, clone D i14, CFT073, and ABU 83972 are in red. ST135 strains NRG 857C and LF82 are in violet. ST127 strain 536 is in pink. ST131 strains uk_P46212, EC958, JJ2434, JJ1887, JJ1886, MNCRE44, ZH193, Ecol_732, CD306, JJ1897, ZH063, SaT040, G749, Ecol_745, Ecol_448, MVAST0167, Ecol_743, and SE15 are in aquamarine. ST15 strain E2348/S69 is in orange. Finally, ST117 strain 2009C-3133 is in gray. (B) Nucleotide pairwise comparison of the MS7163 chromosome to strains UTI89 and S88. Annotated prophages (green) and genomic islands (blue) are indicated. Overall, MS7163 shares highest similarity to S88 with the major difference between the two strains being the presence of phi 2 in MS7163. Download FIG S1, TIF file, 8.1 MB. Copyright © 2018 Nhu et al. 2018 Nhu et al. This content is distributed under the terms of the Creative Commons Attribution 4.0 International license.

10.1128/mBio.01462-18.3 FIG S2  Plasmids of MS7163. (A) Nucleotide pairwise comparison of plasmids pECOS88 (from S88 [top]), pMS7163A (middle), and pUTI89 (from UTI89 [bottom]). Gray shading indicates nucleotide conservation between sequences according to BLASTN (68% to 100%). Overall, pMS7163A shares high sequence conservation with pECOS88 and pUTI89 in the core region (shown in green) containing genes involved in plasmid replication (FIB and FIIA replicons, *sopAB* partition genes, and a *psiAB* toxin-antitoxin system) and conjugation (*tra* genes). The uropathogenicity-associated virulence region in pMS7163A (indicated in blue) was conserved with the corresponding region in pECOS88. This region includes genes encoding factors involved in serum resistance (*iss*), type 1 secretion (*etsABC*), toxicity (*hlyF*), colicin production (colicin Ia, *cia* and *imm*; colicin V, *cvaABC* and *cvi*), and iron acquisition (salmochelin, *iroBCDEN*; aerobactin, *iucABCD* and *iutA*; iron-manganese transport, *sitABCD*). (B) Uropathogenicity-associated virulence-encoding genes located on pMS7163A found in other UPEC chromosomes. Shown is a visualization of the pMS7163A plasmid (the innermost black ring) compared to six UPEC chromosomes using BLASTN, with the cutoff set at 70% nucleotide conservation. Virulence-encoding genes are indicated by the outermost black boxes. The remaining circles represent BLASTN searches against pMS7163A. The table shows the percentage of nucleotide conservation of iron acquisition systems on pMS7163A and on other UPEC chromosomes. (C) Nucleotide pairwise comparison of the multidrug resistance plasmids pR3521 (from UPEC strain EC-3521r [top]), pMS7163B (middle), and pHUSEC41-1 (from EAEC strain HUSEC41 [bottom]). Gray shading indicates nucleotide conservation between sequences according to BLASTN (68% to 100%). Overall, plasmid pMS7163B shares significant nucleotide sequence conservation with pR3521 and pHUSEC41-1 in the core region containing genes associated with plasmid maintenance and conjugation (shown in green). The multidrug resistance region in pMS7163B (indicated in red) is smaller than the corresponding region in the other two plasmids. In pMS7163B, this region contains *sul2* (resistance to sulfonamides) and *dfrA14* (resistance to trimethoprim). Download FIG S2, TIF file, 1.6 MB. Copyright © 2018 Nhu et al. 2018 Nhu et al. This content is distributed under the terms of the Creative Commons Attribution 4.0 International license.

10.1128/mBio.01462-18.8 DATA SET S1  MS7163 genomic analyses and results. This file contains information about mobile genetic elements of MS7163 (including genomic islands and prophages) and the methylome of MS7163. Download DATA SET S1, XLSX file, 0.1 MB. Copyright © 2018 Nhu et al. 2018 Nhu et al. This content is distributed under the terms of the Creative Commons Attribution 4.0 International license.

10.1128/mBio.01462-18.9 DATA SET S2  Supplementary materials used in the study. This file contains E. coli phylogroup B2 complete genomes used in the phylogenetic analysis of MS7163. It also contains strains and primers used in the study. Download DATA SET S2, XLSX file, 0.1 MB. Copyright © 2018 Nhu et al. 2018 Nhu et al. This content is distributed under the terms of the Creative Commons Attribution 4.0 International license.

### Curli mediate biofilm formation and resistance of sessile cells to LL-37.

To investigate the role of curli in virulence, we assessed the ability of MS7163 and MS7163*csgA* to form a biofilm in a microtiter plate assay employing two different growth media. In LB, a condition under which curli are not produced, the ability to form a biofilm was similar for both strains (*P* = 0.0994) (Fig. 2B). In contrast, growth in LB without salt, a condition known to induce curli production, resulted in stronger biofilm formation by MS7163 than MS7163*csgA* (*P* < 0.0001) (Fig. 2B). To specifically investigate how curli-dependent biofilm formation by MS7163 impacts its resistance to LL-37, we allowed MS7163 to form a biofilm by growth in LB broth without salt, separated planktonic (free-swimming) and sessile (biofilm) cells, and tested their susceptibility to LL-37. In these experiments, following a 2-h incubation in the presence of 64 µg/ml of LL-37, most planktonic cells were killed (survival rate of 0.07%), while sessile cells exhibited significant resistance (survival rate of 30.4%; *P* < 0.0001) (Fig. 2C).

### Curli enhance colonization of the mouse urinary tract.

The role of curli in virulence was also assessed by examining the ability of MS7163 and MS7163*csgA* to survive in the mouse urinary tract in a competitive colonization experiment. Mice were coinoculated with MS7163 and MS7163*csgA* strains in a 1:1 ratio. In this mixed infection assay, MS7163 significantly outcompeted MS7163*csgA* in colonization of the bladder (*P* = 0.0078) (Fig. 2D). Together with our biofilm and LL-37 resistance data, these results demonstrate an important role for curli in virulence.

### Large-scale screening for curli-deficient mutants.

We devised a screen based on CR binding in combination with transposon mutagenesis and TraDIS to define the genetic basis of curli production by MS6173 at 37°C. MS7163 was subjected to transposon mutagenesis using a mini-Tn*5* cassette carrying a chloramphenicol resistance gene, and mutants were screened on YESCA-CR agar supplemented with chloramphenicol. Under these conditions, mutants that did not make curli failed to bind CR and were white, mutants that made reduced levels of curli bound CR weakly and were light red, and mutants that were unaltered in their level of curli production bound CR and were red. In total, ~132,000 mini-Tn*5* mutants were screened, from which 71 mutants were identified that were “white” and unable to bind CR and 246 mutants were “light red” and exhibited reduced binding to CR. All of these mutants retained their CR binding phenotype over three successive rounds of subculture on YESCA-CR agar.

### Identification of curli mutants using TraDIS.

The above mutants were examined by TraDIS to enable *en masse* identification of the insertion sites that led to altered CR binding. Analysis of the pooled 246 light red colonies generated 1,004,719 sequence reads that mapped to 105 unique mini-Tn*5* insertion sites on the MS7163 chromosome. These insertion sites were further localized to 22 genes and 6 intergenic regions, and included genes involved in LPS biogenesis (*waaGHILPQ*, *wzxE*, and *rfaH*), regulation (*envZ* and *rpoS*), metabolism (*pgm*, *galE*, *pgi*, and *fbp*), sodium transport (*nhaA*), and septum formation (*nlpD* and *amiB*) (see Table S1 in the supplemental material). Similarly, analysis of the pooled 71 white colonies generated 238,182 sequence reads that mapped to 67 unique mini-Tn*5* insertion sites. These insertion sites were further localized to 19 genes and 3 intergenic regions (Table 1). Among the 19 genes, nine (47.4%) were well characterized with respect to curli production; these included three genes encoding components of the curli assembly machinery (*csgA*, *csgB*, and *csgG*) and six genes associated with curli regulation (*mlrA*, *ihfA*, *ihfB*, *dksA*, *rne*, and *ompR*). The remaining ten genes were novel or poorly characterized with respect to curli synthesis, and thus we focused the remainder of our investigation on these genes.

10.1128/mBio.01462-18.5 TABLE S1  MS7163 genes identified in the transposon mutant screen that were associated with reduced CR binding. Download TABLE S1, XLSX file, 0.1 MB. Copyright © 2018 Nhu et al. 2018 Nhu et al. This content is distributed under the terms of the Creative Commons Attribution 4.0 International license.

**TABLE 1:** MS7163 genes required for curli production

Gene name	No. of inserts	No. of reads	Congo red binding^a^		CsgA expression by defined mutant^a^	Product^b^
			Tn*5* mutants	Defined mutants		
Known curli regulators						
*dksA*	2	4,496	−	ND	ND	RNA polymerase-binding transcription factor
*ihfB*	4	9,172	−	ND	ND	Integration host factor subunit beta
*rne*	1	2,276	−	ND	ND	RNase E
*ihfA*	1	2,120	−	ND	ND	Integration host factor subunit alpha
*mlrA*	9	27,691	−	ND	ND	HTH-type transcriptional regulator
*ompR*	1	3,126	−	ND	ND	Transcriptional regulatory protein
Curli genes						
*csgG*	11	44,759	−	ND	ND	Curli secretion channel
*csgB*	1	2,441	−	ND	ND	Curli minor subunit
*csgA*	3	9,637	−	ND	ND	Curli major subunit
Purine *de novo* biosynthesis						
*purK*	1	2,029	−	−	−	*N*^5^-Carboxyaminoimidazole ribonucleotide synthase
*purF*	2	6,421	−	−	−	Amidophosphoribosyl transferase
*purM*	2	6,906	−	−	−	Phosphoribosylformylglycinamidine cyclo-ligase
*purL*	4	11,161	−	ND	ND	Phosphoribosylformylglycinamide synthase
*purD*	1	1,422	−	−	−	Phosphoribosylamine-glycine ligase
*purH*	2	6,899	−	ND	ND	AICAR transformylase/IMP cyclohydrolase
Intergenic regions						
*yccT-yccU*	2	5,164	−	ND	ND	Intergenic region between *yccT* and *yccU*
*nudE-yrfF*	2	5,713	−	ND	ND	Intergenic region between *nudE* and *yrfF*
*rcpA-gadW*	3	11,579	−	−	−	Upstream of *rcpA* and downstream of *gadW*
Others						
*yrfF*	7	17,067	−	−	−	Putative membrane protein
*kdgK_2*	2	6,169	−	+	+	2-Dehydro-3-deoxygluconokinase
*rcsD*	5	15,895	−	+	+	Phosphotransferase
*wzyE*	1	3,302	−	+	+	ECA polysaccharide chain elongation

a −, negative phenotype; +, positive phenotype; ND, not done.

b HTH, helix-turn-helix; AICAR, 5-aminoimidazole-4-carboxamide ribonucleotide; ECA, enterobacterial common antigen.

### Disruption of genes involved in purine *de novo* biosynthesis prevents curli production.

**FIG 3:**
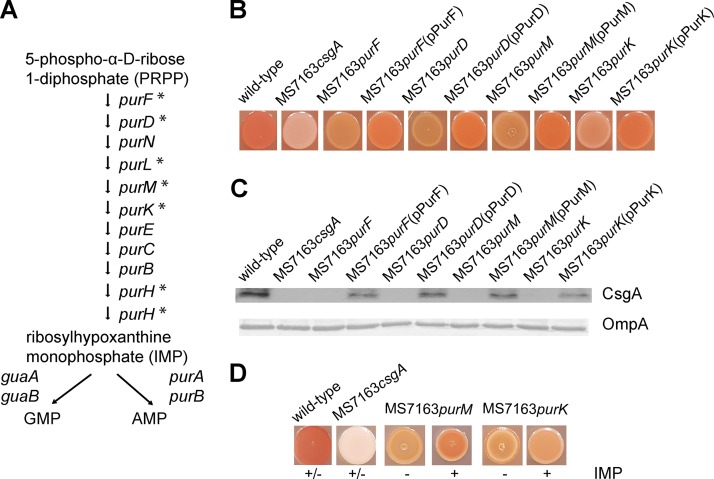
Purine *de novo* biosynthesis is required for curli production. (A) Purine *de novo* biosynthesis pathway in E. coli, adapted from BioCyc. Genes identified by TraDIS that, when mutated, abrogated curli production are indicated by an asterisk. (B) CR staining of wt MS7163, MS7163*csgA*, defined *pur* mutants, and their complemented strains following growth on YESCA-CR agar. Strains were grown by spotting 5 µl of an overnight culture on YESCA-CR agar and incubating the culture for 24 h at 37°C. Mutation of genes in the purine *de novo* biosynthesis abolished CR binding, while complementation restored this phenotype. (C) Western blot analysis of CsgA performed using whole-cell lysates prepared from MS7163 or MS7163*csgA* and *pur* mutants and their complemented strains. Bacteria were grown on YESCA agar for 24 h to induce curli production and treated with formic acid to dissolve polymerized CsgA. Anti-OmpA antibody was used as a loading control. (D) CR staining of wt MS7163, MS7163*csgA*, MS7163*purM*, and MS7163*purK* following growth on YESCA-CR agar with (+) and without (−) IMP supplementation.

Six of the 19 genes identified in our TraDIS analysis (31.6%) form part of the purine *de novo* biosynthesis pathway: i.e., *purF*, *purD*, *purL*, *purM*, *purK*, and *purH* (Table 1). The purine *de novo* biosynthesis pathway comprises 10 genes for the conversion of 5-phospho-α-d-ribose 1-diphosphate (PRPP) to ribosylhypoxanthine monophosphate (IMP), the precursor for AMP and GMP (Fig. 3A). To validate this finding, we examined the involvement of selected purine biosynthesis genes in curli production using defined knockout mutants. Four *pur* mutants were generated by λ-Red-mediated homologous recombination: MS7163*purF*, MS7163*purD*, MS7163*purM*, and MS7163*purK*. Compared to wt MS7163, all four *pur* mutants were unable to bind CR following growth on YESCA-CR agar and failed to express the CsgA major subunit protein based on Western blot analysis of whole-cell lysates employing a CsgA-specific antibody (Fig. 3B and C). Furthermore, complementation of the corresponding gene in each *pur* mutant restored curli production (Fig. 3B and C). Finally, supplementation with IMP, the last product of the purine *de novo* biosynthesis pathway, restored curli production in MS7163*purM* and MS7163*purK* (Fig. 3D). Taken together, these results demonstrate that purine *de novo* biosynthesis plays a critical role in curli production.

### Disruption of *yrfF* prevents curli production.

**FIG 4:**
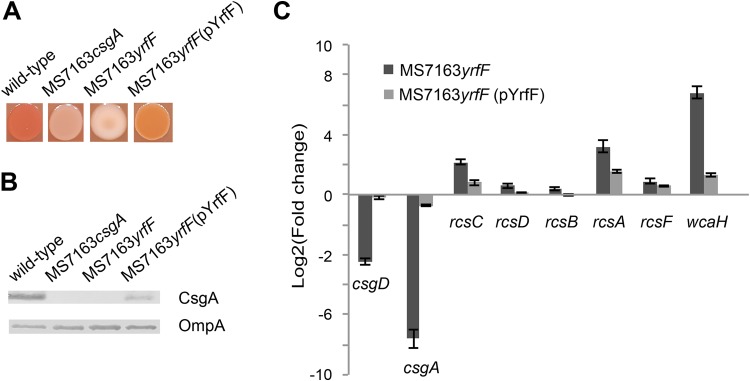
The involvement of the *yrfF* gene in curli production. (A) CR staining of wt MS7163, MS7163*csgA*, MS7163*yrfF*, and the complemented strain MS7163*yrfF*(pYrfF) following growth on YESCA-CR agar. Strains were grown by spotting 5 µl of an overnight culture on YESCA-CR agar and incubating for 24 h. Mutation of *yrfF* abolished CR binding and increased mucoidy, while complementation restored the phenotype to the wild type. (B) Western blot analysis of CsgA performed using whole-cell lysates prepared from MS7163, MS7163*yrfF*, and MS7163*yrfF*(pYrfF). Bacteria were grown on YESCA agar for 24 h to induce curli production and treated with formic acid to dissolve polymerized CsgA. Anti-OmpA antibody was used as a loading control. (C) Impact of *yrfF* on the transcription of genes involved in curli biosynthesis, the Rcs phosphorelay system, and colanic acid production. Shown are the relative fold changes in transcript level of curli genes (*csgD* and *csgA*), Rcs phosphorelay system genes (*rcsCDB-A-F*), and the colanic acid biosynthesis gene *wcaH* in MS7163*yrfF* and the complemented MS7163*yrfF*(pYrfF) strain compared to wt MS7163 as determined by qRT-PCR. Total mRNA was extracted from bacteria grown on YESCA agar at 37°C for 24 h. Results are displayed as the mean log_2_ FC with standard deviation from three independent replicates.

TraDIS analysis identified seven independent insertions within the *yrfF* gene and two insertions immediately upstream of the *yrfF* coding region, strongly suggesting the involvement of YrfF in curli production. YrfF, also known as IgaA in Salmonella enterica, is a negative regulator of the Rcs phosphorelay system, which controls genes involved in colanic acid production (51, 52). Mutation of *yrfF* causes overexpression of the Rcs system, leading to increased colanic acid synthesis (53). To confirm the role of *yrfF* in curli production, we mutated the *yrfF* gene to generate MS7163*yrfF*. MS7163*yrfF* colonies were mucoid and white on YESCA-CR agar, confirming the overexpression of colanic acid and the absence of curli (Fig. 4A). In addition, complementation of MS7163*yrfF* with a plasmid containing the *yrfF* gene (pYrfF) led to loss of mucoidy and restoration of curli production, demonstrated by CR binding and anti-CsgA Western blot analysis (Fig. 4A and B). We also examined the transcription of colanic acid regulatory and biosynthesis genes (*rcsCDB-A-F* and *wcaH*) and selected curli genes (*csgD* and *csgA*) in wt MS7163 compared to MS7163*yrfF* and the complemented strain MS7163*yrfF*(pYrfF). Consistent with our phenotypic data, the transcription of colanic acid genes was increased and curli genes decreased in MS7163*yrfF*, while complementation restored the transcription of these genes to approximately wt level (Fig. 4C). Taken together, these results demonstrate that *yrfF* plays an opposite regulatory role in curli and colanic acid production, respectively.

### Overexpression of the novel regulator RcpA abrogates curli production by repressing the transcription of *csgD* and *csgA*.

**FIG 5:**
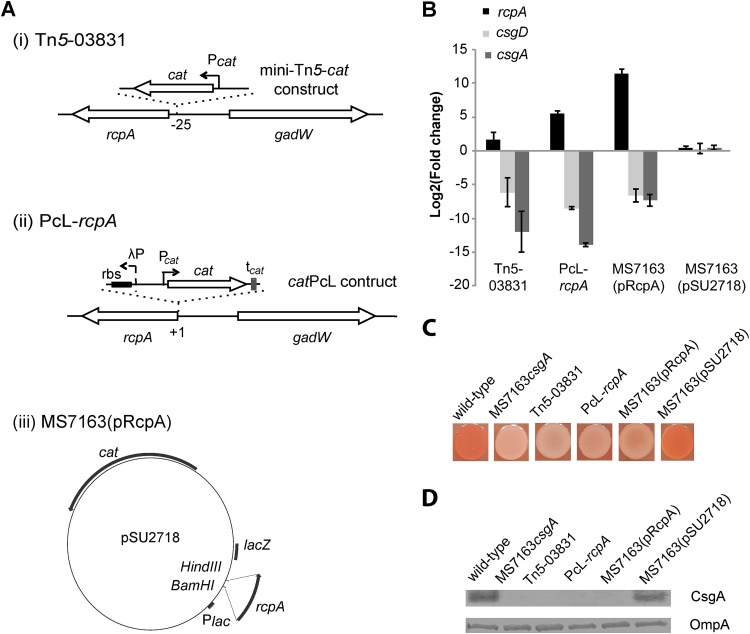
Overexpression of RcpA abolishes curli production. (A) Schematic overview of genetic constructions in *rcpA-*overexpressing mutants. (i) Tn5-03831 mutant, containing the mini-Tn*5* at nucleotide −25 relative to the *rcpA* predicted ATG start codon (P_*cat*_; *cat* gene promoter); (ii) PcL-*rcpA* mutant, PcL promoter and optimized ribosome binding site (rbs) inserted upstream of the *rcpA* CDS; and (iii) plasmid pRcpA, which contains the *rcpA* CDS cloned behind the *lac* promoter in the expression vector pSU2718. (B) Relative fold change in transcript level of *rcpA*, *csgD*, and *csgA* in *rcpA*-overexpressing mutants compared to wt MS7163 as determined by qRT-PCR. Total mRNA was extracted from bacteria grown on YESCA agar at 37°C for 24 h. Results are displayed as the mean log_2_ FC with standard deviation from three independent replicates. (C) CR staining of wt MS7163, MS7163*csgA*, Tn5-03831, PcL-*rcpA*, MS7163(pRcpA), and MS7163(pSU2718 [vector control]) following growth on YESCA-CR agar. Strains were grown by spotting 5 µl of an overnight culture on YESCA-CR agar and incubating for 24 h at 37°C. (D) Western blot analysis of CsgA performed using whole-cell lysates prepared from MS7163, MS7163*csgA*, Tn5-03831, PcL-*rcpA*, MS7163(pRcpA), and MS7163(pSU2718). Bacteria were grown on YESCA agar for 24 h to induce curli production and treated with formic acid to dissolve polymerized CsgA. Anti-OmpA antibody was used as a loading control.

Our TraDIS analysis also identified an enrichment of three independent mini-Tn*5* insertions in the region upstream of a coding sequence (CDS) identified by the locus tag MS7163_03831 (at bp −23, −25, and −26 relative to the MS7163_03831 predicted ATG start codon) (Fig. 5A). In all three cases, the mini-Tn*5* cassette was inserted with the chloramphenicol resistance gene in the same orientation as the MS7163_03831 CDS, referred to henceforth as a repressor of curli production, or *rcpA*. We hypothesized that the mini-Tn*5* insertions led to enhanced transcription of the *rcpA* gene via readthrough from the promoter of the chloramphenicol resistance gene within the mini-Tn*5*, leading to increased expression of RcpA and abolishment of curli production. We tested this by examining the mini-Tn*5* mutant with an insertion at position −25 relative to the predicted *rcpA* start codon (referred to as strain Tn5-03831). Indeed, quantitative reverse transcription-PCR (qRT-PCR) analysis revealed that *rcpA* transcript levels in Tn5-03831 were increased ~3.2-fold compared to that in wt MS7163 (Fig. 5B). In contrast, transcript levels of *csgD* and *csgA* in Tn5-03831 decreased ~70- and ~4,000-fold, respectively. The abolishment of curli production by Tn5-03831 was also confirmed by its inability to bind CR and anti-CsgA Western blot analysis (Fig. 5C and D).

RcpA is a small 282-bp CDS that encodes a putative 93-amino-acid protein of unknown function. To demonstrate that increased expression of RcpA in Tn5-03831 abolished curli production, and rule out the possibility of polar effects or secondary mutations, we generated two types of RcpA-overexpressing strains: (i) PcL-*rcpA*, which contained a strong constitutive promoter (PcL) inserted immediately upstream of the *rcpA* CDS on the MS7163 chromosome and (ii) MS7163(pRcpA), which contained the *rcpA* gene cloned behind the *lac* promoter in plasmid pSU2718 (Fig. 5A). The transcript level of *rcpA* was strongly increased in PcL-*rcpA* and MS7163(pRcpA) compared to wt MS7163 [log_2_ fold changes (FCs) of 5.5 and 11.4 in PcL-*rcpA* and MS7163(pRcpA), respectively] (Fig. 5B). In contrast, the transcription of *csgA* and *csgD* was significantly decreased in both strains [*csgA*, log_2_ FCs of −13.9 and −7.3 in PcL-*rcpA* and MS7163(pRcpA), respectively; *csgD*, log_2_ FCs of −8.5 and −6.6 in PcL-*rcpA* and MS7163(pRcpA), respectively]. Both PcL-*rcpA* and MS7163(pRcpA) did not produce curli based on CR binding and anti-CsgA Western blot analysis (Fig. 5C and D).

### Repression of curli production by RcpA is not strain specific.

**FIG 6:**
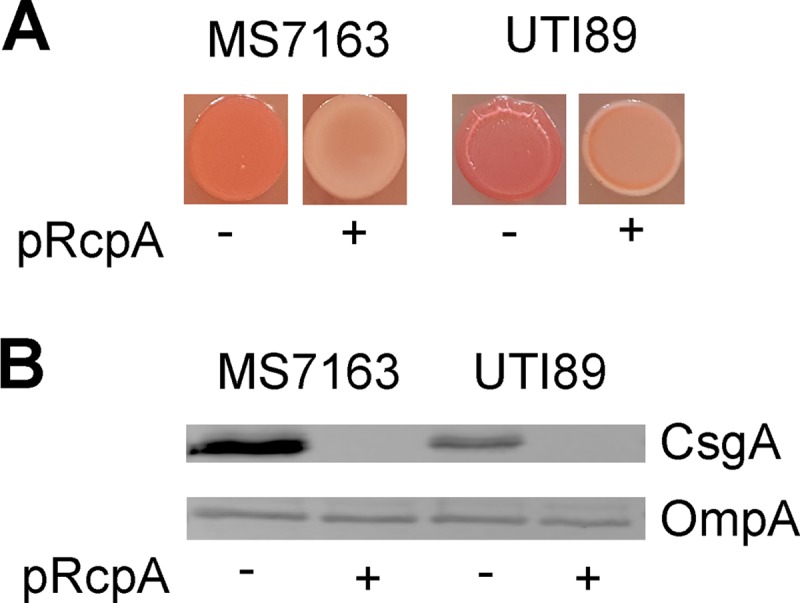
Overexpression of RcpA abolishes curli production in other E. coli strains. (A) CR staining of MS7163 and UTI89 in the absence and presence of plasmid pRcpA following growth on YESCA-CR agar. Strains were grown by spotting 5 µl of an overnight culture on YESCA-CR agar and incubating for 24 h at 37°C. (B) Western blot analysis of CsgA performed using whole-cell lysates prepared from MS7163 and UTI89 in the absence and presence of plasmid pRcpA. Bacteria were grown on YESCA agar for 24 h to induce curli production and treated with formic acid to dissolve polymerized CsgA. Anti-OmpA antibody was used as a loading control.

To extend our findings on the role of RcpA in curli production, we examined the impact of overexpression of RcpA in UPEC strain UTI89, which produces curli during growth at 28°C on YESCA-CR agar (12, 20). Transformation of UTI89 with plasmid pRcpA led to reduced binding to CR (Fig. 6A) and abolishment of CsgA production compared to wild-type UTI89 following growth on YESCA agar (Fig. 6B). Taken together, these results demonstrate that overexpression of RcpA causes repression of curli production, and this function is conserved in different UPEC strains.

### Mutation of *purF* and overexpression of RcpA attenuate colonization of the mouse urinary tract.

**FIG 7:**
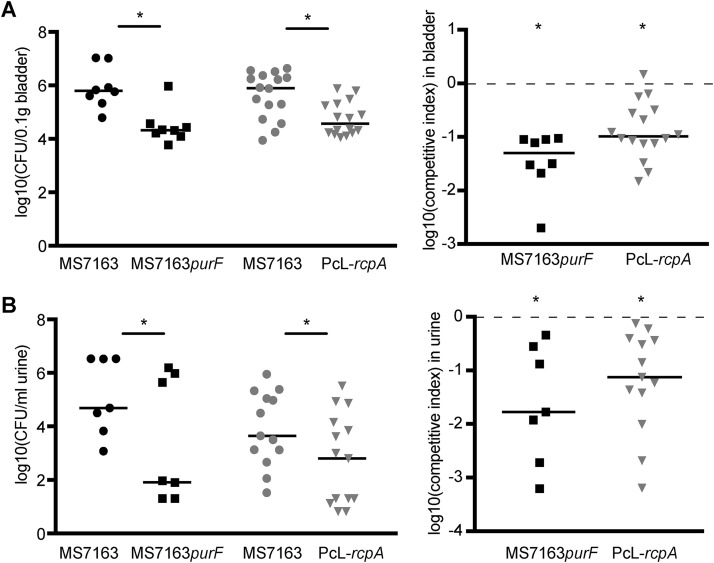
Mutation of *purF* and overexpression of RcpA attenuate colonization of the mouse urinary tract. Competitive mixed-infection mouse experiment employing a 1:1 mixture of MS7163 versus MS7163*purF* and MS7163 versus PcL-*rcpA*. (Left panels) Total CFU enumerated from the bladder (A) and urine (B) of infected mice. Each symbol represents the CFU for each mouse, and the median is represented by a horizontal line. (Right panels) Competitive fitness indices of (A) bladder and (B) urine colonization. Each symbol represents data from an individual mouse in either urine or bladder tissue at 24 h postinfection. Logarithmic values of competitive indices are plotted on the *y* axis; horizontal bars represent median values. A log_10_ fitness index below 0 (shown by the dashed line) indicates that MS7163*purF* and PcL-*rcpA* were at a competitive disadvantage. Statistical significance was determined by the two-tailed Wilcoxon’s signed rank test (*, *P* < 0.05).

Given the impact of mutations in the purine biosynthesis pathway and overexpression of RcpA on curli production, we tested the ability of MS7163*purF* and PcL-*rcpA* to survive in the mouse urinary tract using a competitive infection assay. Female C57BL/6 mice were infected with a 1:1 ratio of MS7163 to MS7163*purF* or MS7163 to PcL-*rcpA* cultured statically in LB without salt, respectively, and colonization was assessed at 1 day postinfection. In these experiments, both the MS7163*purF* and PcL-*rcpA* mutants were significantly outcompeted by wt MS7163 in the bladder and urine of infected mice (Fig. 7). No significant colonization of the kidneys was observed. Taken together, these data demonstrate that disruption of the purine *de novo* biosynthesis pathway and overexpression of RcpA, both of which abrogate curli production, result in attenuated colonization of the mouse urinary tract.

## DISCUSSION

Recent literature has established an increasing appreciation for the role of curli in UPEC virulence (20, 21, 23, 47, 54). These amyloid fibers are produced by many UPEC strains at 37°C, mediate strong biofilm formation, confer resistance to the soluble cationic peptide LL-37, and enhance the production of proinflammatory mediators. Here, we characterized the role of curli in the virulent UPEC strain MS7163 and employed a forward genetic screen to identify new genes involved in the production of curli.

MS7163 was isolated in Slovakia from a patient with severe pyelonephritis (24). Whole-genome sequencing of MS7163 revealed it is most closely related to the neonatal meningitis-causing E. coli (NMEC) strain S88. E. coli S88 was isolated from the cerebrospinal fluid of a neonate and belongs to the highly virulent O45:K1:H7 clone, which accounts for one-third of all neonatal meningitis cases in France (50). Based on *in silico* analysis, MS7163 also possesses this serotype, and both strains share a large plasmid (pMS7163A and pECOS88, respectively) that harbors multiple virulence genes, including three iron uptake systems. Our analysis of the MS7163 genome revealed it contains a mutation in the essential cellulose synthase gene *bcsA* that results in the inability to produce cellulose. Intriguingly, S88 also contains exactly the same mutation, a G deletion at nucleotide 556 of *bcsA* that causes its premature termination. Thus, we speculate that the production of curli, together with the inability to produce cellulose, might represent a previously unrecognized virulence feature of the O45:K1:H7 lineage. Given that the potent immune response stimulated by curli through the activation of interleukin-6 (IL-6) and IL-8 is amplified in the absence of cellulose (21, 23, 31), this phenotype is consistent with our hypothesis. Indeed, a link between the curli-positive, cellulose-negative phenotype and enhanced disease severity has also been demonstrated in the highly virulent E. coli O104:H4 outbreak strain that infected nearly 4,000 people and caused 54 deaths in Germany in 2011 (43).

Growth of MS7163 in LB without salt at 37°C promoted strong biofilm formation, which was abrogated in non-curli-inducing medium (LB broth) and by mutation of the *csgA* gene. We also demonstrated that sessile MS7163 cells were highly resistant to LL-37 compared to their free-swimming planktonic counterpart cells, thus providing direct experimental evidence that curli-mediated biofilm formation significantly enhances LL-37 resistance. LL-37 is a soluble antimicrobial peptide that forms an integral component of the host innate defense system of the urinary tract (23). Thus, curli production would be expected to enhance colonization of the urinary tract, and we demonstrated this in a mixed competitive infection model, where wt MS7163 outcompeted MS7163*csgA* at 24 h postinfection of the mouse bladder. These results were also consistent with previous data demonstrating a role for curli in the early stages (6 h postinfection) of bladder colonization in experimental mice (20).

The application of transposon mutagenesis together with TraDIS represents a powerful combinatorial approach to dissect genetic pathways linked to specific phenotypes (55–57). In this study, we screened ~132,000 mini-Tn*5* mutants by using CR binding following growth on YESCA-CR agar as a proxy for curli production. Based on the size of the MS7163 genome and our previous analysis of unique insertion sites in other TraDIS libraries (55, 57), we estimate this represents a coverage of approximately one insertion every 80 to 100 bp. Overall, two types of mutants were identified: those that were unable to bind CR (white colonies) and those that displayed reduced binding to CR (light red colonies). Using TraDIS, we then mapped the mini-Tn*5* insertion sites of these mutants *en masse*, leading to the identification of 19 genes that were absolutely required for curli production and a further 22 genes that impacted curli production. Many of the genes identified in our screen overlapped with a recent large-scale screen of the E. coli K-12 Keio library (58). In addition to known structural and regulatory genes, these included genes involved in purine biosynthesis, lipopolysaccharide (LPS) biogenesis, stress, and stationary-phase regulation, metabolism, sodium transport, and septum formation. Despite this, we note there were also differences in the total list of genes identified in both studies (see Table S2 in the supplemental material), suggesting strain-specific variation, as well as differences in growth and temperature conditions, may account for divergence in the complex pathway of curli production.

10.1128/mBio.01462-18.6 TABLE S2  Comparative analysis of genes identified to play a role in curli production by MS7163 and the E. coli K-12 strain BW25113. Download TABLE S2, XLSX file, 0.1 MB. Copyright © 2018 Nhu et al. 2018 Nhu et al. This content is distributed under the terms of the Creative Commons Attribution 4.0 International license.

We focused our experiments on genes that were absolutely required for curli production, all of which led to a “white” colony phenotype on YESCA-CR agar. Of the 19 genes identified in this category, three were integral to the curli operon (*csgA*, *csgB*, and *csgG*), and disruption of these genes has previously been shown to abolish curli production in E. coli (25–27, 58–60). We did not identify the curli gene *csgC*; however, this is consistent with other reports that show its mutation does not affect CR binding (26, 29, 58, 61–63). In addition, we did not find insertions in the curli chaperone genes *csgE* and *csgF* in our screen. Mutation of these genes has previously been linked to reduced CR binding, resulting in a light red colony phenotype following growth at ≤28°C on YESCA-CR agar (26, 29, 58, 61–63). It is possible that mutation of these genes in MS7163 led to a subtle change in CR binding phenotype that was missed in our screen or even that there were no insertions in either gene in our library. Interestingly, our screen also failed to identify the *csgD* regulator gene. We generated a targeted *csgD* mutant in MS7163 using λ-Red-mediated homologous recombination and confirmed it does not produce curli (data not shown). Closer examination of *csgD* revealed it contains a lower GC content (41.8%) than the entire MS7163 genome (50.7%), which could explain its absence from our screen as the mini-Tn*5* transposon preferentially inserts into GC-rich regions (64). This has been observed in two previous TraDIS studies, where an increased mini-Tn*5* transposon insertion frequency was detected in high-GC versus low-GC regions (57, 65). Consistent with current knowledge of curli gene control (35, 36, 42), we also identified *mlrA* (which encodes a master regulator of CsgD), *ihfA* and *ihfB* (which together encode IHF), and *ompR* (which encodes a regulator of envelope stress) as positive regulators of curli production, demonstrating their regulatory function is not subjected to temperature control.

In UPEC, purine *de novo* biosynthesis is required for IBC growth in the mouse bladder (66). Our TraDIS screen identified six genes within the purine biosynthesis operon that were required for curli production, and we confirmed the role of four of these genes (*purF*, *purD*, *purM*, and *purK*) by the construction of defined mutants and complementation. We hypothesize that disruption of *de novo* purine biosynthesis results in a decrease in the level of cyclic-di-GMP in the cell. Since cyclic-di-GMP activates the transcription of the *csgD* curli regulator gene (67), this would lead to abolishment of curli production in a *pur*-deficient background. Genes involved in purine biosynthesis were also recently shown to be required for curli production by E. coli K-12 (58), and mutation of *purF* attenuates the ability of UPEC UTI89 to colonize the bladder of C3H/HeN mice (66). Our study demonstrated attenuated bladder colonization by MS7163*purF* in C57BL/6 mice in a mixed competitive colonization experiment. The log_10_ competitive index (CI) of wt MS7163 versus MS7163*purF* (−1.45) was significantly lower than the log_10_ CI of wt MS7163 versus MS7163*csgA* (−0.96; *P* = 0.04), suggesting that the attenuated colonization of MS7163*purF* may be due to the combined effect of reduced fitness via disruption of the purine *de novo* biosynthesis pathway and the inability to produce curli.

The *yrfF* gene was also identified in our screen, and we showed its mutation abolishes curli production. YrfF, known as IgaA in Salmonella enterica serovar Typhimurium, is a negative regulator of the Rcs phosphorelay system, a complex signal transduction pathway that controls the production of colanic acid (51, 52, 68). The Rcs phosphorelay system consists of the histidine sensor kinase RcsC, the phosphoryl group transporter RcsD, and the response regulator RcsB (69). The outer membrane lipoprotein RcsF is also an integral activator of this system and functions by interacting with RcsC and YrfF, both of which are located in the cytoplasmic membrane (51, 52, 70). The *yrfF* gene has been described as essential in several studies (51, 52, 55, 68, 71), as its mutation results in overactivation of RcsCDB and uncontrolled colonic acid biosynthesis (53, 69, 72). This lethal phenotype can be overcome by secondary suppressor mutations in *rcsB*, *rcsC*, and *rcsD*, all of which affect the Rcs system (73). Here, we provide direct evidence for the role of YrfF in curli production through our screen, which identified seven independent insertions in *yrfF* that abrogated CR binding, as well as the construction of a defined mutant (MS7163*yrfF*) and its complementation. We also demonstrated an increase in the transcription of genes in the Rcs system, together with a corresponding decrease in curli gene transcription, in MS7163*yrfF*. This result is consistent with studies in E. coli K-12 that showed the Rcs system negatively controls transcription of the *csg* genes (41). Although *yrfF* was not essential in MS7163, we were unable to test the colonization of MS7163*yrfF* in the mouse bladder, as static culture to enrich for the production of type 1 fimbriae led to spontaneous loss of its mucoid and noncurliated phenotype (see Table S3 in the supplemental material).

10.1128/mBio.01462-18.7 TABLE S3  Instability of MS7163*yrfF* mucoid phenotype following successive rounds of static culture to induce for type 1 fimbria production. Download TABLE S3, XLSX file, 0.1 MB. Copyright © 2018 Nhu et al. 2018 Nhu et al. This content is distributed under the terms of the Creative Commons Attribution 4.0 International license.

We have shown previously that the promoter of the chloramphenicol resistance gene in our mini-Tn*5* transposon can drive the transcription of a downstream gene if the insertion position is favorable (57, 74). This led to the identification of the *rcpA* gene as a novel repressor of curli production. We confirmed this function by overexpression of RcpA using two independent systems: one based on insertion of a constitutive PcL promoter upstream of *rcpA* to drive strong transcription of the gene (PcL-*rcpA*) and a second based on plasmid-mediated overexpression [MS7163(pRcpA)]. In both cases, overexpression of RcpA led to significantly decreased transcription of curli genes (*csgD* and *csgA*), abolishment of CsgA expression, and the inability of the modified strains to bind CR. The PcL-*rcpA* strain was also attenuated in its capacity to colonize the mouse bladder in a mixed infection assay.

The *rcpA* gene encodes a protein 93 amino acids in length. Small proteins, notably those less than 100 amino acids, are frequently ignored due to difficulties in annotation and biochemical detection (75). However, examples of such proteins that localize to the inner membrane and regulate membrane-associated two-component sensor-kinase systems have been described (75). One of the best-characterized small proteins in E. coli is MgrB, a hydrophobic inner membrane protein that functions as a negative-feedback regulator of the PhoP/PhoQ system (76). Analysis of the translated RcpA protein sequence did not identify any known conserved domains or a putative signal sequence. However, using TMHMM2.0 (77), the RcpA protein is predicted to contain two transmembrane helices (amino acids 7 to 26 and 31 to 50) and a cytoplasmic C-terminal domain (amino acids 51 to 93). These properties, in addition to its high hydrophobic amino acid ratio, suggest RcpA may localize to the inner membrane like MgrB. Our analyses showed repression of curli synthesis by overexpression of RcpA was not strain specific and also occurred in UTI89. While the precise mechanism by which RcpA represses curli synthesis remains to be elucidated, we demonstrated its overexpression led to decreased curli gene transcript levels. The *rcpA* gene is conserved in E. coli, *Shigella*, and *Achromobacter*, suggesting its function as a negative regulator of curli synthesis may be broadly conserved.

In summary, this study describes the complete genome sequence of UPEC MS7163, a curli-producing pyelonephritis strain. MS7163 belongs to the highly virulent O45:K1:H7 clone, and we provide the first description of curli production at 37°C by a strain from this lineage. We also describe the identification of several new genes involved in the production of curli and demonstrate their role in UPEC pathogenesis in experimental mice. Further work is now required to understand the precise molecular mechanisms by which these genes impact curli synthesis and the development of UTI.

## MATERIALS AND METHODS

### Ethics statement.

Approval for mouse infection studies was obtained from the University of Queensland Animal Ethics Committee (SCMB/242/16/NHMRC). Experiments were carried out in strict accordance with the recommendations in the Animal Care and Protection Act (Queensland, 2002) and the Australian Code of Practice for the Care and Use of Animals for Scientific Purposes (8th ed., 2013).

### Strains, growth conditions, and genome sequencing.

Bacterial strains and plasmids are listed in Data Set S2. Strains were grown at 37°C on solid or liquid lysogeny broth (LB) medium. To induce curli production, strains were cultured on YESCA agar (10 g/liter Casamino Acids, 1 g/liter yeast extract, 20 g/liter agar) or LB without salt supplemented with 50 µg/ml CR and 1 µg/ml Coomassie brilliant blue as indicated. When required, chloramphenicol (30 µg/ml), gentamicin (20 µg/ml), or IMP (0.5 mM) was added to the medium. Methods for MS7163 genome sequencing and analysis are described in Text S1 in the supplemental material.

10.1128/mBio.01462-18.1 TEXT S1  Methods for MS7163 whole-genome sequencing and analysis. This file contains methods for MS7163 genome sequencing, assembly, annotation, and sequence data analysis. Download TEXT S1, DOCX file, 0.1 MB. Copyright © 2018 Nhu et al. 2018 Nhu et al. This content is distributed under the terms of the Creative Commons Attribution 4.0 International license.

### Transposon mutagenesis and TraDIS.

Generation of the transposon mutant library was performed essentially as previously described (55). MS7163 mini-Tn*5* mutants were plated directly onto YESCA-CR agar supplemented with chloramphenicol and incubated at 37°C for 24 h. Under these growth conditions, CR binding was used as an indicator for curli production: curliated colonies were red, mutants that produced less curli were light red, and mutants that did not produce curli were white. Mutants with an altered CR binding phenotype (i.e., white or light red colonies) were restreaked onto YESCA-CR agar over three successive rounds of subculture to confirm their phenotype and then stored individually in 20% glycerol at −80°C. Multiplex TraDIS analysis of pooled mutants was performed using the Illumina MiSeq platform as previously described (65). Tn*5*-specific reads were identified by FASTX-Toolkit (v.0.0.13) and mapped to the MS7163 complete genome using MAQ (v0.7.1) (78).

### Targeted gene mutation and complementation.

Specific mutants were generated using λ-Red-mediated homologous recombination as previously described (55, 79). Where necessary, the chloramphenicol resistance cassette was removed using plasmid pCP20 (80). Strain PcL-*rcpA* was generated using a previously described methodology (81). Plasmids for complementation were generated by PCR amplification and cloning of genes from MS7163 into plasmid pSU2718 and confirmed by sequencing. All primers are listed in Data Set S2.

### Transcription analysis and Western blotting.

To assess transcript levels of genes involved in curli production, strains were grown on YESCA agar for 24 h at 37°C, harvested directly, resuspended in KPi buffer (28.9 mM KH_2_PO_4_ and 21.1 mM K_2_HPO_4_, pH 7.2) and standardized to an optical density at 600 nm (OD_600_) of 1. A volume of 500 µl of this bacterial suspension was added to 1 ml of RNAprotect bacterial reagent (Qiagen) to stabilize total RNA. RNA extraction, conversion to cDNA, and quantitative reverse transcription-PCR (qRT-PCR) were performed as previously described (82). All primers are listed in Data Set S2. The relative transcript level of target genes from each mutant was compared to wt MS7163 using *gapA* as a control. Relative transcript levels were calculated by the threshold cycle (2^−ΔΔ*CT*^) method (83) and are presented on a log_2_ scale (log_2_ FC) (84). Western blotting for the major curli subunit CsgA was performed following growth in YESCA agar for 24 h at 37°C as described previously (85).

### Biofilm analyses.

Biofilm assays were performed using polyvinyl chloride microplate plates (Corning) and growth in LB or LB without salt with subsequent crystal violet staining as previously described (85). To compare the sensitivity of sessile (biofilm) cells versus planktonic cells to LL-37, MS7163 biofilms were established following growth in LB without salt at 37°C for 24 h. Planktonic cells were removed, and the biofilm was washed 3 times with phosphate-buffered saline (PBS). One set of biofilm cells were incubated in LB for 2 h at 37°C, while another set were incubated in LB containing 64 µg/ml of LL-37 for 2 h at 37°C. After incubation, the biofilm was washed with 1× PBS, cells were detached from the biofilm by repeated pipetting and resuspended in 1× PBS, and the total CFU were enumerated by plating on LB agar. In parallel, planktonic cells were centrifuged, resuspended in either LB or LB containing 64 µg/ml of LL-37, and incubated for 2 h at 37°C, and the total CFU were enumerated by plating on LB agar. The difference in survival rates of planktonic and biofilm cells following incubation with LL-37 was analyzed by *t* test.

### Mouse model of UTI.

The C57BL/6 mouse model of ascending UTI was employed as previously described (16, 86). Strains were enriched for expression of type 1 fimbriae by three successive rounds of static growth in LB without salt for 48 h followed by one round of static growth for 24 h for inoculum preparation and did not display any difference in type 1 fimbria production as assessed by yeast cell agglutination and a *fim* switch orientation PCR (see Fig. S3 in the supplemental material). Curli expression was confirmed by dot blot analysis with CsgA antibody. Infections were performed as competitive assays; the inoculum contained 1:1 strain mixture of wt MS7163 versus MS7163*csgA*, MS7163*purF*, or PcL-*rcpA*. Bacterial loads corresponding to each strain in the urine, bladder, and kidney at 24 hours postinfection (hpi) were enumerated by plating onto LB agar and LB agar supplemented with chloramphenicol. Competitive indices were determined as the ratio of each respective mutant versus wt MS7163 to the ratio of the two strains in the inoculum. Statistical analyses were performed using the two-tailed Wilcoxon matched-pair signed-rank test (Prism7; GraphPad).

10.1128/mBio.01462-18.4 FIG S3  Orientation of the type 1 fimbria phase switch. (A) Map of the phase switch region in both on and off orientations. The promoter (P_*fim*_) is indicated, together with the inverted repeats (IRL and IRR) that flank the invertible segment. The positions of primers P1 and P2 used in the PCR amplification and the sizes of the DNA fragments resulting from HinfI digestion are indicated. (B) Agarose gel showing the fragments obtained by HinfI digestion of the PCR product from MS7163, MS7163*csgA*, MS7163*purF*, and PcL-03831 following static growth in LB broth minus salt. All strains were similarly enriched for bands representing the *fim* switch on orientation (511 bp and 130 bp), suggestive of equivalent type 1 fimbrial expression. Download FIG S3, TIF file, 1.6 MB. Copyright © 2018 Nhu et al. 2018 Nhu et al. This content is distributed under the terms of the Creative Commons Attribution 4.0 International license.

### Accession number(s).

The sequences for the MS7163 chromosome and plasmids pMS7163A and pMS7163B have been deposited in the NCBI GenBank database under GenBank accession no. CP026853, CP026854, and CP026855, respectively. The raw PacBio sequence reads have been deposited in the sequence Read Archive (SRA) under accession no. SRR6727962. The TraDIS reads have been deposited at the SRA under accession no. SRR6706193, SRR6706194, SRR6706195, and SRR6706196.
