# Supplementary figures and images for: Discovery of New Genes Involved in Curli Production by a Uropathogenic Escherichia coli Strain from the Highly Virulent O45:K1:H7 Lineage
Source: mBio. 2018 Aug 21;9(4):e01462-18. doi: 10.1128/mBio.01462-18 (PMC6106082; doi:10.1128/mBio.01462-18)

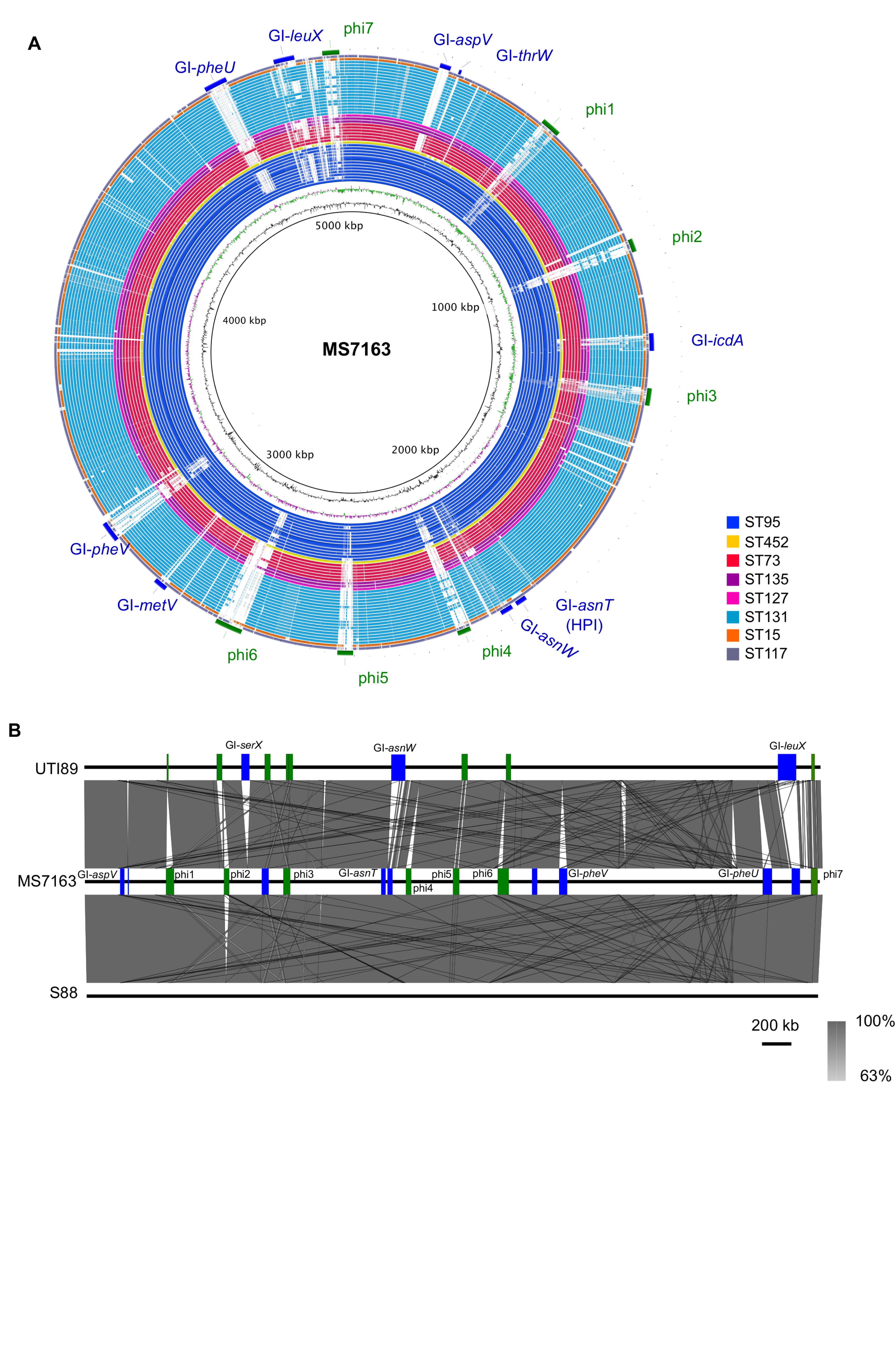

Supplement: FIG S1 [file mbo004184010sf1.tif]

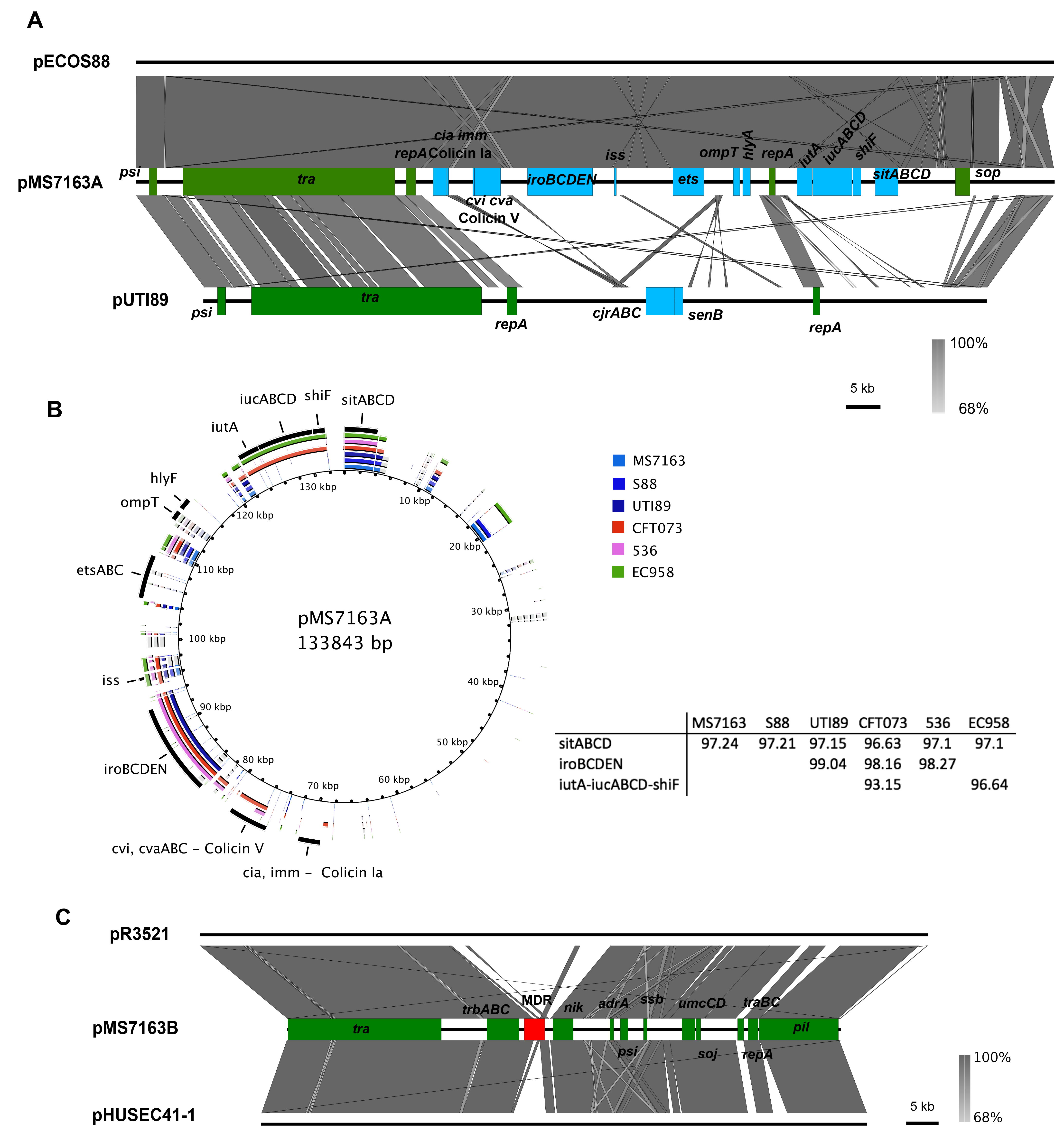

Supplement: FIG S2 [file mbo004184010sf2.tif]

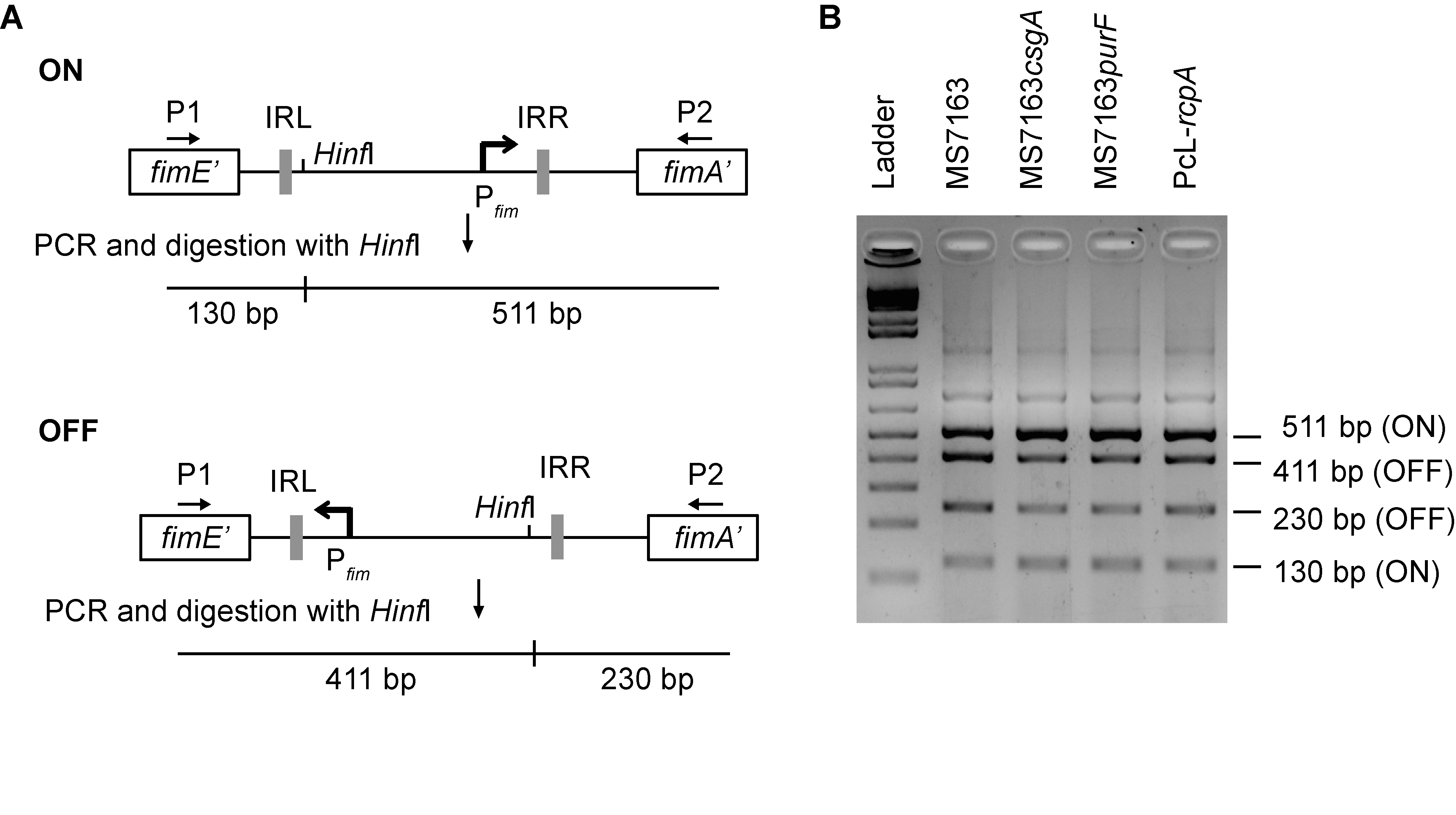

Supplement: FIG S3 [file mbo004184010sf3.tif]
