# Supplementary material for: Discovery of New Genes Involved in Curli Production by a Uropathogenic Escherichia coli Strain from the Highly Virulent O45:K1:H7 Lineage
Source: mBio. 2018 Aug 21;9(4):e01462-18. doi: 10.1128/mBio.01462-18 (PMC6106082; doi:10.1128/mBio.01462-18)
Supplement: TEXT S1 [file mbo004184010s1.docx]

**Methods for whole genome sequencing and data analysis.**

MS7163 genomic DNA was extracted using Ultraclean**^®^** Microbial DNA Isolation Kit (MO BIO) according to the manufacturer’s instructions. The genome of MS7163 was sequencing on a PacBio RS II sequencing instrument using 3 SMRT cells, a 10 kb insert library and the P6-C4 sequencing chemistry. *De novo* genome assembly was performed using PacBio's SMRT Portal (v2) and the hierarchical genome assembly process (HGAP v2.0) with default settings and a seed read cut-off length of 5 kb. Assembly resulted in 5 contigs, three representing the chromosome and two completely assembled plasmids. The three chromosomal contigs were then aligned to the most closely related phylogroup B2 *E. coli* complete genomes using Contiguity (1). Contig gaps were manually closed using the raw PacBio sequence data and verified by PCR with primers MS7163gap-F and MS7163gap-R (File S2). The complete genome sequence of MS7163 (a chromosome and 2 plasmids) was annotated using Prokka (2) and insertion sequence (IS) annotation was done with ISFinder (<https://www-is.biotoul.fr/>). Annotation of CDS and IS was then curated manually. Uropathogenic related virulence genes and antibiotic resistance genes were analyze using VFDB (Virulence factor database) (3, 4) and ResFinder 2.1 (5), respectively. Comparative genomics of MS7163 genomes and other UPEC genomes were performed using BLAST (6), BRIG (7), ACT (8) and Easyfig (9). Phylogenetic relationship of MS7163 and other UPEC strains was analyzed using kSNP v.2 (10). *In silico* MLST typing was performed using the Achtman scheme with seven housekeeping genes (11).

**References**

1. **Sullivan MJ, Ben Zakour NL, Forde BM, Stanton-Cook M, Beatson SA.** 2015. Contiguity: Contig adjacency graph construction and visualisation. PeerJ PrePrints **3**:e1037v1.

2. **Seemann T.** 2014. Prokka: rapid prokaryotic genome annotation. Bioinformatics **30**:2068-9.

3. **Chen L, Xiong Z, Sun L, Yang J, Jin Q.** 2012. VFDB 2012 update: toward the genetic diversity and molecular evolution of bacterial virulence factors. Nucleic Acids Res **40**:D641-5.

4. **Chen L, Yang J, Yu J, Yao Z, Sun L, Shen Y, Jin Q.** 2005. VFDB: a reference database for bacterial virulence factors. Nucleic Acids Res **33**:D325-8.

5. **Zankari E, Hasman H, Cosentino S, Vestergaard M, Rasmussen S, Lund O, Aarestrup FM, Larsen MV.** 2012. Identification of acquired antimicrobial resistance genes. J Antimicrob Chemother **67**:2640-4.

6. **Altschul SF, Gish W, Miller W, Myers EW, Lipman DJ.** 1990. Basic local alignment search tool. J Mol Biol **215**:403-10.

7. **Alikhan NF, Petty NK, Ben Zakour NL, Beatson SA.** 2011. BLAST Ring Image Generator (BRIG): simple prokaryote genome comparisons. BMC Genomics **12**:402.

8. **Carver T, Berriman M, Tivey A, Patel C, Bohme U, Barrell BG, Parkhill J, Rajandream MA.** 2008. Artemis and ACT: viewing, annotating and comparing sequences stored in a relational database. Bioinformatics **24**:2672-6.

9. **Sullivan MJ, Petty NK, Beatson SA.** 2011. Easyfig: a genome comparison visualizer. Bioinformatics **27**:1009-10.

10. **Gardner SN, Hall BG.** 2013. When whole-genome alignments just won't work: kSNP v2 software for alignment-free SNP discovery and phylogenetics of hundreds of microbial genomes. PLoS ONE **8**:e81760.

11. **Wirth T, Falush D, Lan R, Colles F, Mensa P, Wieler LH, Karch H, Reeves PR, Maiden MC, Ochman H, Achtman M.** 2006. Sex and virulence in Escherichia coli: an evolutionary perspective. Mol Microbiol **60**:1136-51.
